# 3D Printed SiOC(N) Ceramic Scaffolds for Bone Tissue Regeneration: Improved Osteogenic Differentiation of Human Bone Marrow-Derived Mesenchymal Stem Cells

**DOI:** 10.3390/ijms222413676

**Published:** 2021-12-20

**Authors:** Yuejiao Yang, Apoorv Kulkarni, Gian Domenico Soraru, Joshua M. Pearce, Antonella Motta

**Affiliations:** 1 BIOtech, Center for Biomedical Technologies, University of Trento, Via Sommarive 9, 38123 Trento, Italy; antonella.motta@unitn.it; 2 European Institute of Excellence on Tissue Engineering and Regenerative Medicine Unit, Via delle Regole 101, 38123 Trento, Italy; 3 Glass & Ceramics Lab, Department of Industrial Engineering, University of Trento, Via Sommerive 9, 38123 Trento, Italy; giandomenico.soraru@unitn.it; 4 Department of Electrical and Computer Engineering, Western University, 1151 Richmond St. N., London, ON N6A 5B9, Canada; joshua.pearce@owu.ca

**Keywords:** bone tissue regeneration, polymer derived ceramics, biocompability, stem cells, osteogenic differentiation, additive manufacturing, fused filament fabircation, cellular ceramics, open source 3D printing

## Abstract

Bone tissue engineering has developed significantly in recent years as there has been increasing demand for bone substitutes due to trauma, cancer, arthritis, and infections. The scaffolds for bone regeneration need to be mechanically stable and have a 3D architecture with interconnected pores. With the advances in additive manufacturing technology, these requirements can be fulfilled by 3D printing scaffolds with controlled geometry and porosity using a low-cost multistep process. The scaffolds, however, must also be bioactive to promote the environment for the cells to regenerate into bone tissue. To determine if a low-cost 3D printing method for bespoke SiOC(N) porous structures can regenerate bone, these structures were tested for osteointegration potential by using human mesenchymal stem cells (hMSCs). This includes checking the general biocompatibilities under the osteogenic differentiation environment (cell proliferation and metabolism). Moreover, cell morphology was observed by confocal microscopy, and gene expressions on typical osteogenic markers at different stages for bone formation were determined by real-time PCR. The results of the study showed the pore size of the scaffolds had a significant impact on differentiation. A certain range of pore size could stimulate osteogenic differentiation, thus promoting bone regrowth and regeneration.

## 1. Introduction

Silicon-based ceramic scaffolds for bone regeneration are one of the main strategies used for the treatment of bone loss and large-scale bone defects [1,2,3,4,5]. Current studies show that silicon is an essential element for bone development and formation [6,7]. Silicon-based materials play an important role in the surface bioactivity through the exchange of ions at the scaffold–tissue interface, which results in the formation of a layer, similar to the mineral phase of bone [8]. Silicon possesses similar properties to phosphorus when it comes to bone formation and development [9]. Previous research has shown that the expression of some osteogenesis-related genes, e.g., alkaline phosphatase (ALP), bone morphogenetic protein-2(BMP-2), and collagen type I (Col I), are affected by silicon [10]. Silicon also contributes to the promotion of early deposition of apatite, the growth of osteoblasts, and some genes that control the induction of cell cycles and progression which enhance osteogenesis [11]. Enhanced apatite mineralization ability, biocompatibility, and bioactivity are also seen. A significant increase in the expression of angiogenic factors of human bone marrow-derived mesenchymal stem cells (BMSCs), osteogenic genes, and alkaline phosphatase activity were seen due to the presence of silicon [12].

Moreover, the silicon-based ceramic scaffolds for bone regeneration have a highly porous 3D architecture with interconnected porosities, which is a similar architecture to that of bone. These porosities allow cells to adhere, proliferate, migrate and invade the vasculature [13,14]. The pore size, however, can be tailored for the targeted tissue and the cells, as the size of the pores and the density significantly affect cell adhesion, proliferation, and spatial distribution [15,16]. It has been shown that the osteogenic differentiation of human mesenchymal stem cells (hMSCs) was highly dependent on the geometry of the scaffolds [17,18]. Along with the porosity, the scaffolds should also have similar mechanical properties to the bone in which the scaffold is to be implanted. Since porosity can act against the mechanical strength, the scaffolds need to have the right balance of porosity and mechanical properties [19,20]. Some studies have shown that the best possible properties for scaffolds would be comparable to the cortical bone, where the ideal porosity would be around 60–90%, with a pore size greater than 150 microns [21,22,23]. Undesirable mechanical properties of scaffolds can lead to undesirable results during osteogenic differentiation. Thus, it is preferable to have scaffold materials with properties similar to the bones [24,25,26].

Thanks to the development of additive manufacturing technology, the complex structure and various pore sizes of the silicon-based ceramic scaffold can be easily obtained. Various 3D printing methods have showcased the fabrication of ceramic scaffolds, such as selective laser sintering (SLS) [27,28], stereolithography (SLA) [29], binder jetting, material jetting, etc. The simplest, most widespread and lowest-cost method of 3D printing is material extrusion or fused filament fabrication (FFF) [30,31]. Direct (material) extrusion of ceramics, however, is limited to certain ceramics that can be made into slurries or inks/gels, and the resolution and details obtained from them are not adequate for many applications. Polymer-based FFF, on the other hand, has been undergoing rapid open source technical evolution [32,33] and resolutions better than 100 microns can be achieved [34].

Polymer-derived ceramics, PDCs, are novel multifunctional ceramics obtained from pyrolysis in a controlled atmosphere of preceramic polymers [35]. PDCs can be processed in different shapes including porous foams and aerogels [36,37]. PDCs have already been showcased for biomedical applications such as multidrug delivery systems [38,39]. A novel method of fabricating SiOC(N) cellular structures with dense struts by integrating FFF 3D printing with polymer-derived ceramics was reported in our previous paper. The method utilizes the simplicity of the polymer FFF combined with the polymer-derived ceramics to obtain high-resolution ceramic scaffolds [40,41] with both tunable pore size and low-cost fabrication on a RepRap-class 3D printer able to print thermoplastic elastomer [42,43]. Moreover, the preliminary biological evaluations in a previous study showed that the ceramic material has good cytocompatibility, promoting fast cell adhesion and early-stage cell activities [40]. This makes it possible to apply the scaffold to mimic the bone tissue geometry and microenvironment for bone regeneration. 

The study presents in vitro testing of the SiOC(N) porous ceramic scaffolds for bone regeneration applications. C eramic scaffolds with two different pore sizes (300 µm and 500 µm) were tested here to demonstrate the impact of the pore size on cell behavior. Human bone marrow-derived mesenchymal stem cells (hMSCs) were seeded on the top of the scaffolds and cultured in the osteogenic differentiation medium for 21 days. The cell metabolism, proliferation, alkaline phosphatase (ALP) activity, and morphology were evaluated. Finally, the gene expression level of the main osteogenic differentiation markers was investigated by quantitative real-time PCR.

## 2. Materials and Methods

### 2.1. Preparation of Ceramic Scaffolds

**Figure 1:**
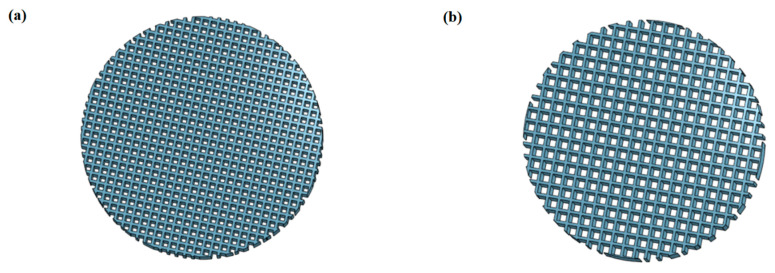
(**a**) 3D representation of the 300-micron pore size test sample; (**b**) 3D representation of the 500-micron pore size test sample.

The samples were designed [44] (OnShape, PTC) as porous discs of 13 mm diameter and 1.5 mm thickness. The samples were designed with different pore sizes of 300 microns (small pores) and 500 microns (large pores) to analyze the effect of pore size on bioactivity (Figure 1). They were first printed on an open source RepRap-class FFF 3D printer (Lulzbot TAZ 6, Fargo Additive Manufacturing Equipment 3D, Fargo, ND, USA) using NinjaFlex TPU with a 0.15 mm nozzle. The scaffolds were printed with the pore sizes of 500 and 700 microns since the pyrolysis process results in 25–30% shrinkage. They were then impregnated with a solution of acetone, polysilazane preceramic polymer (Durazane 1800, Merck Gmbh, Darmstadt, Germany), and catalyst platinum divinyltetramethyldisiloxane complex, Pt 2% in xylene (CAS number: 68478-92-2, Sigma-Aldrich, St. Louis, MO, USA). The discs were then dried for 24 h in the air and pyrolyzed in a tube furnace (Gero tube furnace) at 1200 °C for 1 h in a nitrogen atmosphere with 400 cc/min flow. The obtained scaffolds were first rinsed with deionized water (DI water) and then sterilized by autoclave at 121 °C for 15 min.

### 2.2. Silicon Release

The release of silicon ions was tested by submerging the samples in PBS solution (EuroClone). The samples were submerged into 5 mL of PBS solution and kept in the incubator at 37 °C. At each time point, a 2 mL solution was extracted and replaced by a fresh PBS solution. The extracted solution was tested for silicon ions using ion coupled plasma employing optical emission spectroscopy (ICP-OES) (SPECTRO Analytical Instruments, Kleve, Germany). Eight replicates were used for each pore size at each time point. The final results were calculated as the total released amount.

### 2.3. Cell Culture

Human bone marrow-derived mesenchymal stem cell line (hMSCs, ATCC number: PCS-500-012) was cultured in *α*-MEM medium supplemented with 10% Fetal Bovine Serum (FBS, EuroClone) and 1% Antibiotic/Antimycotic (AA, EuroClone), in a humidified atmosphere of 5% CO_2_ at 37 °C. The medium was changed every two days. Once they reached 70% confluence, the cells were detached by 1% trypsin-EDTA solution, counted, and re-suspended in standard medium with the concentration of 500,000 cells/mL.

### 2.4. Cell Seeding and Differentiation

After placing sterilized samples into 24-well plates, 0.6 mL cell suspensions (standard medium) were added directly to the samples (300,000 cell/well) and tissue culture plates (TCP), which were the control group (only cells without samples). The plates were incubated in a humidified atmosphere of 5% CO_2_ at 37 °C to promote cell adhesion. Twenty-four hours after the seeding, the samples with cells were moved to new plates and the medium was switched into the differentiation medium: standard medium with 0.1 µM dexamethasone (DEX), 0.1 mM ascorbic acid 2-phosphate (AP) and 10 mM β-Glycerophosphate (BGP). The differentiation medium was changed every two days until 21 days.

### 2.5. AlamarBlue Assay

The cells’ viability activity on different pore sizes (300 μm and 500 μm) after 7 days, 14 days, and 21 days of culture was determined with AlamarBlue Cell Viability assay (Invitrogen, Carlsbad, CA, USA), which quantifies cellular metabolic activity. AlamarBlue reagent was added directly to each well at 10% of the cell culture medium volume. Then, the well plates were incubated at 37 °C in a humidified atmosphere with 5% CO_2_ for 3 h. From each well, 100 μL of the solution was collected. The fluorescence signal was measured with a Tecan Infinite 200 microplate reader (Tecan Group, Männedorf, Switzerland) with an excitation wavelength of 560 nm and an emission wavelength of 590 nm. TCP was used as the control group and eight replicates were considered for each experimental condition.

### 2.6. DNA Quantification Assay

To evaluate cell proliferation on the different pore sizes (300 μm and 500 μm), a PicoGreen DNA quantification assay (Quant-iT PicoGreen dsDNA Assay, Invitrogen, Carlsbad, CA, USA) was used. TCP was used as the control group. After 7 days, 14 days, and 21 days of culture, the culture medium was removed, and the samples were washed with PBS. Samples were then covered with 300 μL of 0.05% Triton-X PBS solution and incubated at 37 °C for 1 h. Before analysis, the samples were sonicated for 10 s with a Hielscher ultrasonic homogenizer (UP400S, 400 W-24 kHz, cycle 1, amplitude 40%, from Hielscher Ultrasonics, Teltow, Germany). Subsequently, 100 μL supernatant of each sample was placed in a black 96-well plate and mixed with 100 μL of PicoGreen working solution, prepared following the manufacturer’s instructions. Fluorescence intensity was measured with a Tecan Infinite 200 microplate reader (Tecan Group, Männedorf, Switzerland) using an excitation wavelength of 485 nm and an emission wavelength of 535 nm. A calibration curve was created using a double-stranded DNA standard provided by the kit and was used for the calculation of the DNA content. Finally, the approximate number of cells per sample was determined from DNA content by the conversion factor of 7.7 pg DNA per cell. Eight replicates were considered for each pore size at each time point.

### 2.7. Alkaline Phosphatase (ALP) Activity Assay

ALP activity of the cells on scaffolds with different pore sizes was evaluated by the Alkaline Phosphatase assay kit (abcam, Cambridge, UK). The preparation procedure of the samples is the same as for the DNA quantification assay, in which the supernatant was obtained after washing, incubating, and sonication. Non-fluorescent 4-methylumbelliferyl phosphate disodium salt (MUP) substrate and MUP reaction solution were prepared following the instructions of the manufacturer. The reaction wells were set up in a black 96-well plate by mixing supernatant with MUP reaction solution and stop solution, using the volume suggested by the instruction. A standard curve was also created using the ALP enzyme. The fluorescence intensity was measured at excitation wavelength 485 nm and emission wavelength 535 nm. The readings of the samples were applied to the standard curve to obtain the amount of MUP generated by the ALP sample, and the activity of ALP in the tested samples was calculated by dividing the amount of 4-MU by the volume of the sample. Eight replicates were considered for each pore size at each time point.

### 2.8. RNA Isolation

Total mRNA was isolated from the scaffolds directly by NucleoZLO reagent (MACHEREY-NAGEL, Düren, Germany) according to the protocol from the manufacturer. The isolated RNA of the samples was dissolved in 20 μL RNase-free water; the final concentration of RNA was determined by a NanoDrop (ND-1000 Spectrophotometer, Thermo Fisher Scientific, Waltham, MA, USA) and then diluted into 10 ng/μL.

### 2.9. cDNA Synthesis

The isolated RNA was reverse transcribed into cDNA by iScropt Reverse Transcription Supermix kit (BIO-RAD, Hercules, CA, USA). Then, 4 μL iScript RT Supermix was mixed with 16 μL of isolated RNA (total RNA 160 ng) for each reverse transcription reaction well. Then, the complete reaction mix was incubated in Bio-Rad CFX96 Touch (BIO-RAD, Hercules, CA, USA) using a thermal cycling protocol provided by the manufacturer.

### 2.10. Gene Expression by Quantitative Real-Time PCR (RT-qPCR)

The quantification of gene expression was performed by Bio-Rad CFX96 Touch (BIO-RAD, USA). SsoAdvanced Universal SYBR Green Supermix kit was used, and the primer assays used in this study were listed in Table 1. The tested samples were mixtures that consisted of 5 μL SsoAdvanced Universal SYBR Green Supermix, 0.5 μL primer, and 5 μL cDNA sample, which led to a final amount of 40 ng cDNA per well. The PCR amplification was carried out as follows: polymerase activation and DNA denaturation at 95 °C for 42 s, followed by 40 cycles at 60 °C and 30 s for each cycle. Then, the melt curve was performed between 95 to 65 °C with 0.5 °C increments at 2 to 5 s/step. The PCR results were relatively quantified with the comparative ΔΔC_T_ method by CFX Manager Software, comparing to the housekeeping mRNA expression of glyceraldehyde-3 phosphate dehydrogenase (GAPDH). The analysis of each gene was processed in duplicate and there were eight replicates for each sample. 

### 2.11. Cell Morphology, Distribution, and Immunofluorescence Staining

Cell morphology and distribution were visualized by Oregon green phalloidin and 4′6-diamidino-2-phenylindole (DAPI) staining. Oregon green phalloidin stains actin filaments of cytoskeleton resulting in green fluorescence while DAPI stains nuclei resulting in blue fluorescence. After 7 days, 14 days, and 21 days of culture, the cell-seeded samples were fixed with 4% paraformaldehyde, washed three times with PBS, and then were permeabilized using 0.2% Triton X-100 PBS solution for 30 min. After washing with PBS 3 times (15 min each time), cells were incubated in Oregon green phalloidin (5.0 μL/well) and DAPI (1.0 mL/well, 5.4 μL dilute in 25.0 mL PBS) solution for 1 h at room temperature. After three rinses with PBS, samples were observed using Zeiss LSM 510 Meta confocal laser scanning microscope.

### 2.12. Statistical Analysis

GraphPad Prism 9 (La Jolla, CA, USA) was used for statistical analysis for all the data obtained from each independent experiment. Where applicable, data were expressed as mean ± SD. The statistical analysis was performed by two-way ANOVA using the all-pair-wise multiple comparison procedure, in which * *p* < 0.05 were set as the level of significance.

## 3. Results 

### 3.1. Structural Characterization of SiOC(N) Ceramic Scaffolds

**Figure 2:**
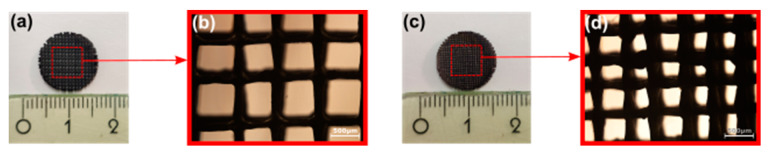
Large (**a**) and small (**c**) pore size SiOC(N) scaffold; the scaffold structure under the optical microscope (**b**,**d**). The scale bars in (**b**,**d**) are 500 μm.

Two different pore sizes (500 μm and 300 μm) (Figure 2) were selected to mimic the pore size of human bone. The theoretical surface area of the porous discs was calculated using OnShape and the results are shown in Table 2. The structure of the final products was observed by an optical microscope. Theoretically, the total surface of discs with small pores is 1.26 times larger and the top/bottom surface is 1.36 times larger, compared to the total surface area of discs with large pores.

### 3.2. Silicon Iron Release of SiOC(N) Ceramic Scaffolds

**Figure 3:**
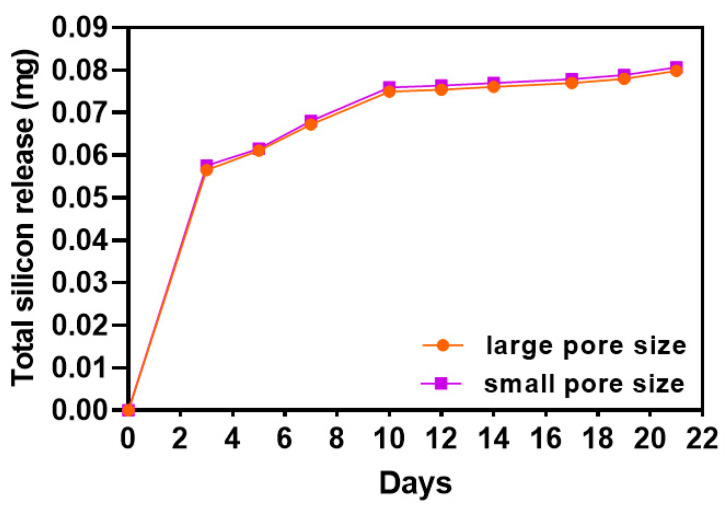
Silicon ion release curve. *n* = 8.

The release of silicon ion was monitored for 21 days by ICP-OES, and the total amount of the released silicon ion was calculated and plotted in Figure 3. Scaffolds with large pore size and small pore size had almost the same releasing curve and amount. Until day 10, the amount of released silicon kept increasing, and after 10 days of release, the released amount of Si ion reached a plateau. 

### 3.3. Characterization of Proliferation, Metabolism of hMSCs during Osteogenic Differentiation

**Figure 4:**
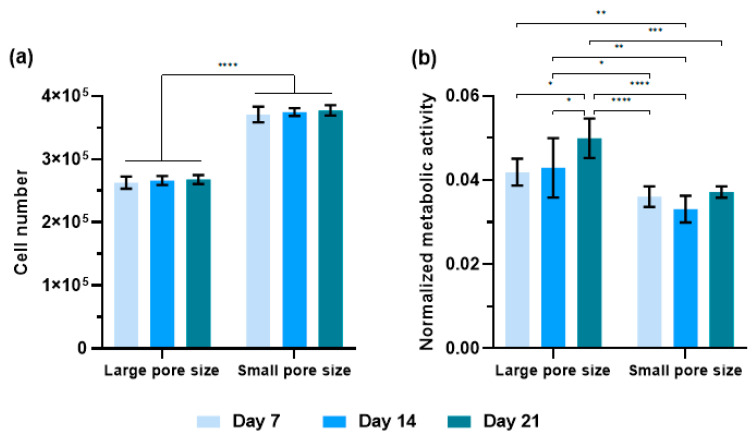
(**a**) Cell proliferation and (**b**) Metabolic activity of hMSCs in 7 days, 14 days, and 21 days of cell culture. *n* = 8; * *p* < 0.05; ** *p* < 0.01; *** *p* < 0.001; **** *p* < 0.0001.

Cell proliferation and metabolic activity of hMSCs seeding on SiOC(N) ceramic scaffolds with different pore sizes were performed on day 7, day 14, and day 21 in the presence of the osteogenic medium, using PicoGreen DNA quantification assay and AlamarBlue assay, respectively. The results are shown in Figure 4. The small pore size scaffolds induced a higher proliferation rate than the large ones. Interestingly, the cell number did not change over time in all the groups during 21 days of culture. Metabolic activity was normalized by cell numbers for each group at each time point. Except for day 7, in which the data of large and small pore size scaffolds showed no significant difference, cells cultured on the large pore size scaffold showed, in general, a higher metabolism activity than those cultured of small pore size scaffold, in particular on day 21.

### 3.4. Visualization of Cell Morphology and Distribution by Confocal Laser Scanning Microscopy

**Figure 5:**
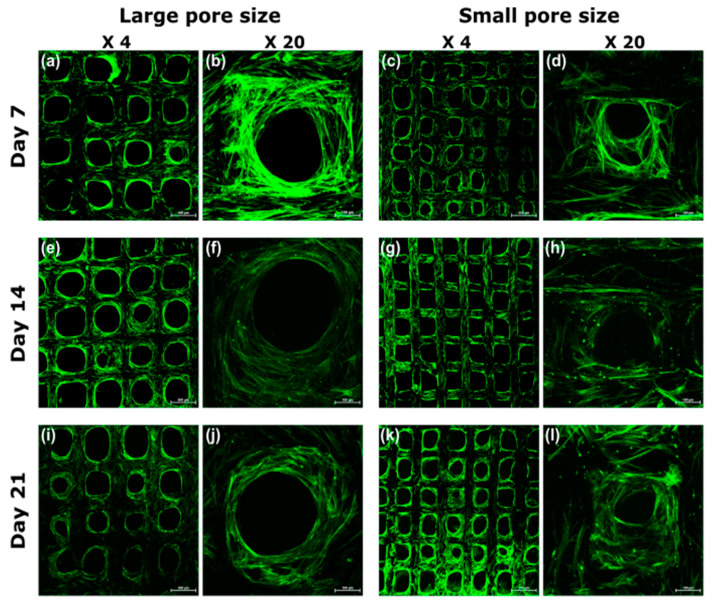
(**a**–**l**) Confocal images of hMSCs morphology on the scaffolds with different pore sizes cultured in the osteogenetic medium for 7, 14, and 21 days. Each sample was presented in low (×4) and high (×20) magnification. The scale bars in the images of ×4 columns are 500 μm, and the scale bars in the images of ×20 columns are 100 μm.

The confocal images were taken in both low and high magnification on scaffolds with different pore sizes (Figure 5). Cell morphologies during the osteogenic differentiation were evaluated by staining the cell cytoskeleton with Oregon green phalloidin (green). In general, the scaffolds could promote very good cell adhesion during the cell culture period. In the first week, the adhered cells morphology exhibited a high degree of spreading with many cell/cell connections due to filopodia and lamellipodia formation (Figure 5b,d) in all groups, which was even more evident on small pore size scaffolds (Figure 5d). Instead, at 2–3 weeks of culture, adhered cells of all the groups penetrated in the pores, colonizing the 3D structure. A difference in cell behavior, however, was observed during the 3 weeks of culture. Despite a large number of cells on the scaffolds, the spatial organization of cells was quite different. On the large pore size scaffolds, the cells were aligning on the surfaces, but were not able to cross the empty space. However, the cellular structure of the small pore size scaffold was small enough for the cells to bridge the pore and the pores were almost filled by cells over time. 

### 3.5. Characterization of ALP Activity of hMSCs during Osteogenic Differentiation

**Figure 6:**
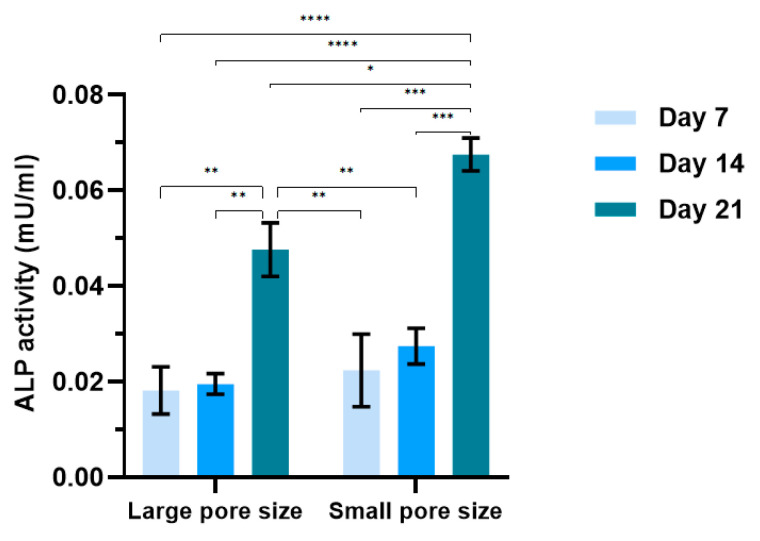
ALP activity of hMSCs on large and small pore size SiOC(N) ceramic scaffold in osteogenic medium for 7, 14, and 21 days. *n* = 8; * *p* < 0.05; ** *p* < 0.01; *** *p* < 0.001; **** *p* < 0.0001.

ALP production is widely used as a marker of bone cells because it is associated with osteoblastic differentiation. In this work, ALP activity was measured by the Alkaline Phosphatase assay kit, and the results are presented in Figure 6. In the first two weeks of differentiation, both groups had similar levels of ALP activity. A dramatic increase in the ALP activity occurred on day 21, which was more than two times higher than on day 7 and day 14, which is particularly relevant on the small pore size scaffold on day 21.

### 3.6. Expression of Osteogenic Marker Genes in the Presence of the SiOC(N) Ceramic Scaffold

**Figure 7:**
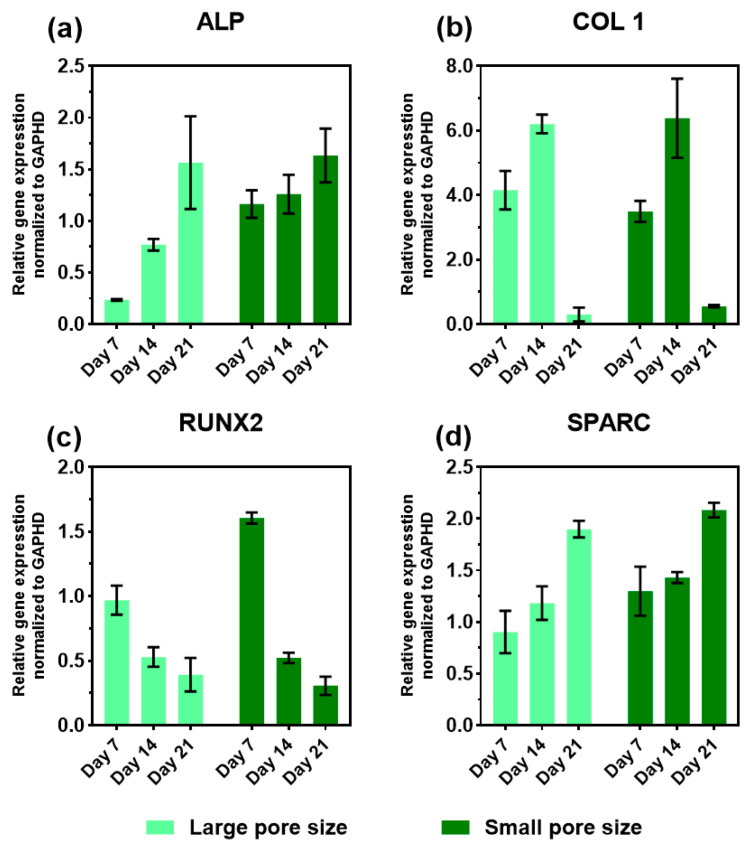
Relative gene expression of ALP (**a**), COL 1 (**b**), RUNX2 (**c**), and SPARC (**d**) of hMSCs on SiOC(N) ceramic scaffolds with different pore sizes after culturing in osteogenic medium for day 7, 14, and 21. TCP with hMSCs was used as a reference group. GAPDH was used as a housekeeping gene. *n* = 8.

**Figure 8:**
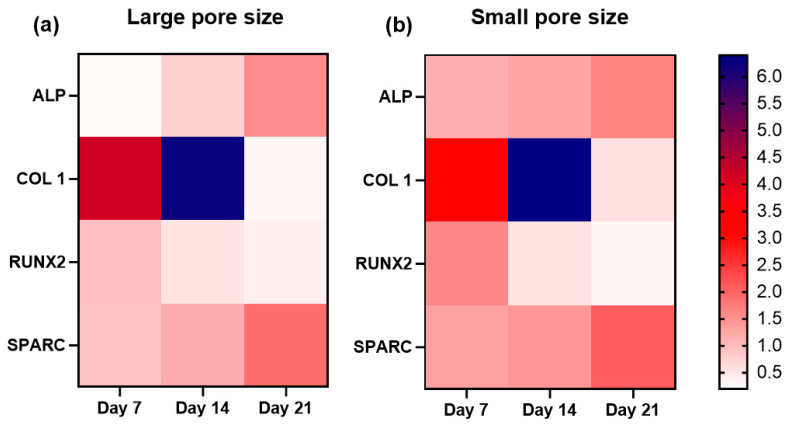
Heat map of gene expression of small (**a**) and large (**b**) pore size SiOC(N) ceramic scaffolds. *n* = 8.

The relative mRNA expression levels of the osteogenic markers: alkaline phosphatase (ALP), collagen type I (COL 1), runt-related transcription factor 2 (RUNX2), and osteonectin (SPARC) were monitored on day 7, 14, and 21 using RT-qPCR. The total RNA was isolated from cells seeded on SiOC(N) scaffolds with different pore sizes and cultured in the osteogenic medium. The results were presented in Figure 7 and the heat map of the gene expression overview were presented in Figure 8. 

Generally, the gene expression trends of the selected markers were similar in both sample groups (Figure 7) and these trends were different from TCP during the 21 days of culture (Supplementary Materials, Figure S1). An increasing level of ALP expression was observed in both groups. It should be noted that the ALP expression of the small pore size scaffold was significantly higher at the first experimental time point (day 7), compared with the large pore size samples. Additionally, both groups reached a similar expression level of ALP on day 21. The expression of COL 1 for both groups showed a peak on day 14 and dropped almost to zero on day 21. No significant differences showed in the expression of COL 1 during 21 days between the two groups at the same time point. A downregulation of RUNX2 was observed in both groups during the three weeks, but the small pore size scaffold showed a higher level in the first week compared with the large pore size group. The expression of osteonectin (SPARC), for both groups, showed the same trend along with the three weeks of culture and similar levels.

## 4. Discussion

Tissue engineering is based on the use of instructive scaffolds that should provide 3D templates for initial cell attachment, proliferation, and subsequent tissue formation. Therefore, the materials used as well as the geometry of the scaffold have relevant impacts on the biological performance. In our previous study [40], the fabrication procedures were optimized and the physical, mechanical, chemical, and preliminary biological properties of the SiOC(N) ceramic scaffolds were already thoroughly characterized. The results demonstrated that the novel method eliminates the possibility of having pores or defects inside the struts with complete impregnation of the preceramic polymer in the TPU structures. Another issue with biomedical implant structures is that they are very expensive to manufacture. The research also showed that the scaffolds can be manufactured, with high reproducibility and industrial tolerances, at an affordable and thus accessible price. The preliminary biological studies proved the material to be non-cytotoxic, promote fast cell adhesion, and early-stage cell activations [40]. In the present study, the research is focused on the impact of the geometry of the scaffold on the osteogenic differentiation of hMSCs. 

Different biological results were obtained by comparing two groups of scaffolds that are different in total surface areas and pore sizes, and, according to the literature, these differences can affect the releasing amount of silicon ions. In the current work, despite the scaffold with the small pore size having a larger surface area (26% more) the results of silicon ion release showed that there is no significant difference in the release amount between the two groups (Figure 3). This suggests that the only parameter that causes differences in cell behaviors is the pore size (in terms of morphology, proliferation, metabolic activity, ALP activity, and gene expression).

The ability of the materials to induce good cell adhesion and proliferation was confirmed with a higher cell number on small pore size scaffolds due to the larger surface area available. It is interesting to observe that, for both groups, cell numbers (Figure 4a) from one week to three weeks remained constant, demonstrating that the cells were differentiating in the very early stage (first week). As reported in the literature [45], during the differentiation process, cell proliferation and differentiation are two interdependent processes that have a counteracting relationship, and this correlated with the obtained results. Cell spatial organization, detected by confocal images (Figure 5), shows that the cells on the small pore size scaffolds were able to build a 3D network, migrating into the structure and connecting to each other crossing the pores (Figure 5d,h,i). The 3D cell distribution is a more physiological cell organization, promoting stem cell differentiation into the bone cells [46]. When the pore size is too large, the 3D structure is not able to drive cells forming the 3D organization, slowing down the differentiation. In fact, this is confirmed by the gene expression (Figure 7) that the cells on small pore size scaffolds were able to differentiate in an earlier stage, particularly in the expression of ALP (Figure 7a) and RUNX2 (Figure 7c).

The geometry differences in the scaffolds also had an impact on the cell activities during the osteogenic differentiation. In the presented work, general and phenotypic-specific metabolism were determined by AlamarBlue assay and ALP production, respectively. It is reported in the literature that an increase in ALP activity should be associated with osteoblastic differentiation. ALP is thought to increase and then decrease when mineralization is well progressed [47]. The cells on small pore size scaffolds showed a lower general metabolic activity (Figure 4b), but higher ALP activity (Figure 6) compared to cells on large pore size scaffolds, especially on day 21. The significant increase in this enzyme between the second and third week suggested that cells on small pore size scaffolds were shifting to a more differentiated state. 

The mRNA levels of osteogenic genes (ALP, COL 1, RUNX2, and SPARC) were evaluated during the 21 days of differentiation, using RT-qPCR. The positive impact of Si-based materials on osteogenic differentiation has been investigated in numerous studies [48,49], in which the water-soluble silicon was proved to enhance osteoblast proliferation and differentiation under in vitro conditions. The investigated expression levels of osteoblast-specific marker genes in terms of osteoblast differentiation, have shown a special pattern that could help to have a perception of the functional bone construct (Figure 8). RUNX2, a member of the runt homology domain transcription factor family, plays a crucial role in osteoblast development. It is a major gene responsible for the early orientation of stem cells towards osteoblastic lineage and directly activates the transcription of genes such as osteocalcin, COL 1, and ALP. Moreover, it is typically downregulated in three or four weeks of culture, which is an important indicator of matrix maturation and mineralization [50]. In fact, there was a significant decrease in the expression of RUNX2 observed in both groups during the second and the third week, suggesting the acceleration of the differentiation. COL 1 plays an important role in biomineralization. It is expressed in high levels near the end of the proliferation state and during the period of matrix deposition. It is also known to decrease with time and an ongoing calcification of the bone tissue [51]. The increase in COL 1 expression showed a peak on day 14 and then dropped to a very low level in both scaffolds between the second and the third week. This observation is in accordance with the expression profiles reported in the literature for the osteogenic differentiation of hMSCs [52]. The ALP gene is first detected in osteoblast progenitor cells which are committed to differentiate into osteoblasts. The expression of ALP is dependent on the maturation stages of osteoblasts. It increases when mineralization is well progressed and then decreases in later stages [53]. In this case, upregulation of ALP expression was observed in both scaffolds in 21 days. Although, the expression level showed significant differences on day 21 between the samples with large pore size and small pore size. It is worth noting that hMSCs on small pore size expressed a significantly high level of ALP on day 7 compared to that on large pore size, suggesting the cells on the small pore size scaffolds had an earlier differentiation. Osteonectin (SPARC) is a phosphorylated glycoprotein that plays a role in regulating the initiation, and promotion of mineralization as well as crystal growth [54]. In this work, it is revealed that the expression of osteonectin is increased in hMSCs during differentiation on both groups, this may be due to the osteoprogenitor culture conditions. 

## 5. Conclusions

In summary, the bioactivity and osteogenic differentiation ability of this open-source 3D printing process for SiOC(N) ceramic scaffolds with different pore sizes was investigated by in vitro cell culture of hMSCs. The results showed that the material of the scaffold has a good ability to release water-soluble silicon ions and the total amount of released silicon ions was not affected by the pore size. Moreover, the scaffolds were able to improve the differentiation while retaining the cell number. It has been demonstrated that the pore size of the scaffold has a strong impact on cell behaviors including cell number, metabolism, ALP activity, distribution, and the speed of osteogenic differentiation. This open-source 3D printing process for SiOC(N) ceramic scaffolds is promising and provides opportunities to have more complex and precise structural matrices with controllable bioactivity for bone regeneration applications. Future work, including in vivo testing, is warranted in order to improve the technology to be used as bone implants. 

## Figures and Tables

**Table 1:** Selected primers for gene expression.

Code	Gene	Primer	Catalog No.
ALP	Alkaline phosphatase	ALPL, human	qHsaCID0010031
COL1	Collagen type I	COL1A1, human	qHsaCED0043248
RUNX2	Runt-related transcription factor 2	RUNX2, human	qHsaCED0044067
SPARC	Osteonectin	SPARC, human	qHsaCID0010332
GAPHD	glyceraldehyde-3 phosphate dehydrogenase	GAPDH, human	qHsaCED0038674

**Table 2:** Sample information.

	Large Pore Size	Small Pore Size
Diameter (mm)	13	13
Thickness (mm)	1.5	1.5
Pore size (μm)	500	300
Theoretical total surface area (mm^2^)	1077.00	1358.84
Theoretical top/bottom surface area (mm^2^)	53.88	73.81

## Supplementary Materials

The following are available online at www.mdpi.com/article/10.3390/ijms222413676/s1.

## References

[1] Hing K.A., Revell P.A., Smith N., Buckland T. (2006). Effect of Silicon Level on Rate, Quality and Progression of Bone Healing within Silicate-Substituted Porous Hydroxyapatite Scaffolds. Biomaterials.

[2] Casarrubios L., Gómez-Cerezo N., Sánchez-Salcedo S., Feito M.J., Serrano M.C., Saiz-Pardo M., Ortega L., de Pablo D., Díaz-Güemes I., Fernández-Tomé B. (2020). Silicon Substituted Hydroxyapatite/VEGF Scaffolds Stimulate Bone Regeneration in Osteoporotic Sheep. Acta Biomater..

[3] Kamboj N., Aghayan M., Rodrigo-Vazquez C.S., Rodríguez M.A., Hussainova I. (2019). Novel Silicon-Wollastonite Based Scaffolds for Bone Tissue Engineering Produced by Selective Laser Melting. Ceram. Int..

[4] Du X., Fu S., Zhu Y. (2018). 3D Printing of Ceramic-Based Scaffolds for Bone Tissue Engineering: An Overview. J. Mater. Chem. B.

[5] Baino F., Fiume E. (2020). 3D Printing of Hierarchical Scaffolds Based on Mesoporous Bioactive Glasses (MBGs)—Fundamentals and Applications. Materials.

[6] Arora M., Arora E. (2017). The Promise of Silicon: Bone Regeneration and Increased Bone Density. J. Arthrosc. Jt. Surg..

[7] Götz W., Tobiasch E., Witzleben S., Schulze M. (2019). Effects of Silicon Compounds on Biomineralization, Osteogenesis, and Hard Tissue Formation. Pharmaceutics.

[8] Amaral M., Costa M.A., Lopes M.A., Silva R.F., Santos J.D., Fernandes M.H. (2002). Si3N4-Bioglass Composites Stimulate the Proliferation of MG63 Osteoblast-like Cells and Support the Osteogenic Differentiation of Human Bone Marrow Cells. Biomaterials.

[9] Li R., Ying B., Wei Y., Xing H., Qin Y., Li D. (2020). Comparative Evaluation of Sr-Incorporated Calcium Phosphate and Calcium Silicate as Bioactive Osteogenesis Coating Orthopedics Applications. Colloids Surf. A Physicochem. Eng. Asp..

[10] Gomes P.S., Botelho C., Lopes M.A., Santos J.D., Fernandes M.H. (2010). Evaluation of Human Osteoblastic Cell Response to Plasma-Sprayed Silicon-Substituted Hydroxyapatite Coatings over Titanium Substrates. J. Biomed. Mater. Res. B Appl. Biomater..

[11] Liu X., Xie Y., Ding C., Chu P.K. (2005). Early Apatite Deposition and Osteoblast Growth on Plasma-Sprayed Dicalcium Silicate Coating. J. Biomed. Mater. Res. Part A.

[12] Wang X., Zhou Y., Xia L., Zhao C., Chen L., Yi D., Chang J., Huang L., Zheng X., Zhu H. (2015). Fabrication of Nano-Structured Calcium Silicate Coatings with Enhanced Stability, Bioactivity and Osteogenic and Angiogenic Activity. Colloids Surf. B Biointerfaces.

[13] Zhang C., Hu Y.-Y., Cui F.-Z., Zhang S.-M., Ruan D.-K. (2006). A Study on a Tissue-Engineered Bone Using RhBMP-2 Induced Periosteal Cells with a Porous Nano-Hydroxyapatite/Collagen/Poly(l-Lactic Acid) Scaffold. Biomed. Mater..

[14] Barrère F., Mahmood T.A., de Groot K., van Blitterswijk C.A. (2008). Advanced Biomaterials for Skeletal Tissue Regeneration: Instructive and Smart Functions. Mater. Sci. Eng. R Rep..

[15] Zeltinger J., Sherwood J.K., Graham D.A., Müeller R., Griffith L.G. (2001). Effect of Pore Size and Void Fraction on Cellular Adhesion, Proliferation, and Matrix Deposition. Tissue Eng..

[16] O’Brien F.J., Harley B.A., Yannas I.V., Gibson L.J. (2005). The Effect of Pore Size on Cell Adhesion in Collagen-GAG Scaffolds. Biomaterials.

[17] Kuboki Y., Jin Q., Takita H. (2001). Geometry of Carriers Controlling Phenotypic Expression in BMP-Induced Osteogenesis and Chondrogenesis. J. Bone Jt. Surg..

[18] Hulbert S.F., Young F.A., Mathews R.S., Klawitter J.J., Talbert C.D., Stelling F.H. (1970). Potential of Ceramic Materials as Permanently Implantable Skeletal Prostheses. J. Biomed. Mater. Res..

[19] Chen G., Dong C., Yang L., Lv Y. (2015). 3D Scaffolds with Different Stiffness but the Same Microstructure for Bone Tissue Engineering. ACS Appl. Mater. Interfaces.

[20] Breuls R.G.M., Jiya T.U., Smit T.H. (2008). Scaffold Stiffness Influences Cell Behavior: Opportunities for Skeletal Tissue Engineering. Open Orthop. J..

[21] Mastrogiacomo M., Muraglia A., Komlev V., Peyrin F., Rustichelli F., Crovace A., Cancedda R. (2005). Tissue Engineering of Bone: Search for a Better Scaffold. Orthod. Craniofacial Res..

[22] Chan B.P., Leong K.W. (2008). Scaffolding in Tissue Engineering: General Approaches and Tissue-Specific Considerations. Eur. Spine J..

[23] Hollister S.J. (2005). Porous Scaffold Design for Tissue Engineering. Nat. Mater.

[24] Boccaccio A., Ballini A., Pappalettere C., Tullo D., Cantore S., Desiate A. (2011). Finite Element Method (FEM), Mechanobiology and Biomimetic Scaffolds in Bone Tissue Engineering. Int. J. Biol. Sci..

[25] Kelly D.J., Prendergast P.J. (2006). Prediction of the Optimal Mechanical Properties for a Scaffold Used in Osteochondral Defect Repair. Tissue Eng..

[26] Sandino C., Lacroix D. (2011). A Dynamical Study of the Mechanical Stimuli and Tissue Differentiation within a CaP Scaffold Based on Micro-CT Finite Element Models. Biomech. Model Mechanobiol..

[27] Williams J.M., Adewunmi A., Schek R.M., Flanagan C.L., Krebsbach P.H., Feinberg S.E., Hollister S.J., Das S. (2005). Bone Tissue Engineering Using Polycaprolactone Scaffolds Fabricated via Selective Laser Sintering. Biomaterials.

[28] Xia Y., Zhou P., Cheng X., Xie Y., Liang C., Li C., Xu S. (2013). Selective Laser Sintering Fabrication of Nano-Hydroxyapatite/Poly--ε-Caprolactone Scaffolds for Bone Tissue Engineering Applications. IJN.

[29] Kim K., Yeatts A., Dean D., Fisher J.P. (2010). Stereolithographic Bone Scaffold Design Parameters: Osteogenic Differentiation and Signal Expression. Tissue Eng. Part B Rev..

[30] Zein I., Hutmacher D.W., Tan K.C., Teoh S.H. (2002). Fused Deposition Modeling of Novel Scaffold Architectures for Tissue Engineering Applications. Biomaterials.

[31] Do A.-V., Khorsand B., Geary S.M., Salem A.K. (2015). 3D Printing of Scaffolds for Tissue Regeneration Applications. Adv. Healthc. Mater..

[32] Jones R., Haufe P., Sells E., Iravani P., Olliver V., Palmer C., Bowyer A. (2011). RepRap—The Replicating Rapid Prototyper. Robotica.

[33] Bowyer A. (2014). 3D Printing and Humanity’s First Imperfect Replicator. 3D Print. Addit. Manuf..

[34] Zhang B., Seong B., Nguyen V., Byun D. (2016). 3D Printing of High-Resolution PLA-Based Structures by Hybrid Electrohydrodynamic and Fused Deposition Modeling Techniques. J. Micromech. Microeng..

[35] Colombo P., Mera G., Riedel R., Sorarù G.D. (2010). Polymer-Derived Ceramics: 40 Years of Research and Innovation in Advanced Ceramics. J. Am. Ceram. Soc..

[36] Santhosh B., Vakifahmetoglu C., Ionescu E., Reitz A., Albert B., Sorarù G.D. (2020). Processing and Thermal Characterization of Polymer Derived SiCN(O) and SiOC Reticulated Foams. Ceram. Int..

[37] Vakifahmetoglu C., Semerci T., Gurlo A., Soraru G.D. (2021). Polymer Derived Ceramic Aerogels. Curr. Opin. Solid State Mater. Sci..

[38] Arango-Ospina M., Xie F., Gonzalo-Juan I., Riedel R., Ionescu E., Boccaccini A.R. (2020). Review: Silicon Oxycarbide Based Materials for Biomedical Applications. Appl. Mater. Today.

[39] Vakifahmetoglu C., Zeydanli D., Ozalp V.C., Borsa B.A., Soraru G.D. (2018). Hierarchically Porous Polymer Derived Ceramics: A Promising Platform for Multidrug Delivery Systems. Mater. Des..

[40] Kulkarni A., Pearce J., Yang Y., Motta A., Sorarù G.D. (2021). SiOC(N) Cellular Structures with Dense Struts by Integrating Fused Filament Fabrication 3D Printing with Polymer-Derived Ceramics. Adv. Eng. Mater..

[41] Kulkarni A., Sorarù G.D., Pearce J.M. (2020). Polymer-Derived SiOC Replica of Material Extrusion-Based 3-D Printed Plastics. Addit. Manuf..

[42] Woern A.L., Pearce J.M. (2017). Distributed Manufacturing of Flexible Products: Technical Feasibility and Economic Viability. Technologies.

[43] Xiao J., Gao Y. (2017). The Manufacture of 3D Printing of Medical Grade TPU. Prog. Addit. Manuf..

[44] Open-Source Foundation. https://osf.io/grv8m/.

[45] Stein G.S., Lian J.B., Owen T.A. (1990). Relationship of Cell Growth to the Regulation of Tissue-Specific Gene Expression during Osteoblast Differentiation. FASEB J..

[46] Bicer M., Cottrell G.S., Widera D. (2021). Impact of 3D Cell Culture on Bone Regeneration Potential of Mesenchymal Stromal Cells. Stem Cell Res. Ther..

[47] Mayer H., Bertram H., Lindenmaier W., Korff T., Weber H., Weich H. (2005). Vascular Endothelial Growth Factor (VEGF-A) Expression in Human Mesenchymal Stem Cells: Autocrine and Paracrine Role on Osteoblastic and Endothelial Differentiation. J. Cell. Biochem..

[48] Nair M.B., Bernhardt A., Lode A., Heinemann C., Thieme S., Hanke T., Varma H., Gelinsky M., John A. (2009). A Bioactive Triphasic Ceramic-Coated Hydroxyapatite Promotes Proliferation and Osteogenic Differentiation of Human Bone Marrow Stromal Cells. J. Biomed. Mater. Res. Part A.

[49] Botelho C.M., Brooks R.A., Best S.M., Lopes M.A., Santos J.D., Rushton N., Bonfield W. (2006). Human Osteoblast Response to Silicon-Substituted Hydroxyapatite. J. Biomed. Mater. Res. Part A.

[50] Komori T. (2006). Regulation of Osteoblast Differentiation by Transcription Factors. J. Cell. Biochem..

[51] Amiri B., Ghollasi M., Shahrousvand M., Kamali M., Salimi A. (2016). Osteoblast Differentiation of Mesenchymal Stem Cells on Modified PES-PEG Electrospun Fibrous Composites Loaded with Zn2SiO4 Bioceramic Nanoparticles. Differentiation.

[52] Zur Nieden N.I., Kempka G., Ahr H.J. (2003). In Vitro Differentiation of Embryonic Stem Cells into Mineralized Osteoblasts. Differentiation.

[53] Aubin J.E., Triffitt J.T., Bilezikian J.P., Raisz L.G., Rodan G.A. (2002). Chapter 4—Mesenchymal Stem Cells and Osteoblast Differentiation. Principles of Bone Biology.

[54] Fuchs S., Jiang X., Schmidt H., Dohle E., Ghanaati S., Orth C., Hofmann A., Motta A., Migliaresi C., Kirkpatrick C.J. (2009). Dynamic Processes Involved in the Pre-Vascularization of Silk Fibroin Constructs for Bone Regeneration Using Outgrowth Endothelial Cells. Biomaterials.

