# Supplementary material for: 3D Printed SiOC(N) Ceramic Scaffolds for Bone Tissue Regeneration: Improved Osteogenic Differentiation of Human Bone Marrow-Derived Mesenchymal Stem Cells
Source: Int J Mol Sci. 2021 Dec 20;22(24):13676. doi: 10.3390/ijms222413676 (PMC8706922; doi:10.3390/ijms222413676)
Supplement: Supplementary file 1 [file ijms-22-13676-s001.zip › ijms-1493219-supplementary.pdf]

International Journal of
Molecular Sciences

Supplementary Materials

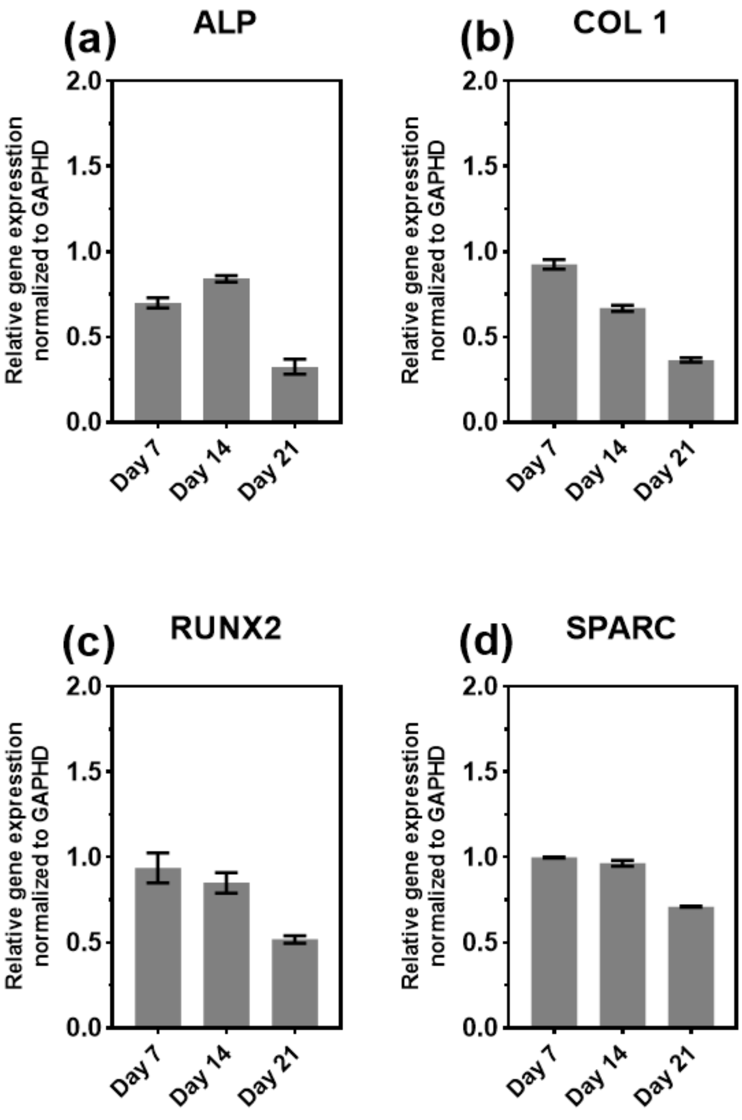

Figure S1. Relative gene expression of ALP (**a**), COL 1 (**b**), RUNX2 (**c**), and SPARC (**d**) of hMSCs on tissue culture plate (TCP) after culturing in osteogenic medium for day 7, 14, and 21. TCP with hMSCs was used as a reference group. GAPDH was used as a housekeeping gene.
